# Glucagon-like peptide-1 receptor agonists and advanced liver outcomes in type 2 diabetes: a systematic review and exploratory meta-analysis

**DOI:** 10.3389/fendo.2026.1874720

**Published:** 2026-06-02

**Authors:** Jing-Hong Hu, Ming-Ling Chang, Tung-Jung Huang, Nai-Jen Liu, Yung-Yu Hsieh

**Affiliations:** 1 Division of Gastroenterology and Hepatology, Yunlin Chang Gung Memorial Hospital, Yunlin, Taiwan; 2 Division of Gastroenterology and Hepatology, Linkou Chang Gung Memorial Hospital & College of Medicine, Chang Gung University, Taoyuan, Taiwan; 3 Division of Pulmonary and Critical Care Medicine, Yunlin Chang Gung Memorial Hospital, Yunlin, Taiwan; 4 Division of Gastroenterology, Chiayi Chang Gung Memorial Hospital, Chiayi, Taiwan

**Keywords:** comparative effectiveness research, diabetes mellitus, type 2, glucagon-like peptide 1 receptor agonists, hepatocellular carcinoma, liver cirrhosis, metabolic dysfunction-associated steatotic liver disease, real-world evidence

## Abstract

Glucagon-like peptide-1 receptor agonists (GLP-1RAs) improve weight, glycemic control, and cardiometabolic risk factors, but randomized trials have not been powered to determine effects on advanced liver outcomes. We conducted a systematic review and exploratory meta-analysis of peer-reviewed comparative real-world cohort studies in adults with type 2 diabetes (T2D), emphasizing active-comparator designs, clinically advanced liver endpoints, and transparent limits of causal inference. PubMed/MEDLINE and Embase were searched with citation chasing and an updated PubMed/MEDLINE search through 25 April 2026; this evidence-identification strategy was focused rather than exhaustive. Twelve eligible comparative studies were included. In the predefined active-comparator outcome-family stratum, four studies comparing GLP-1RAs with DPP-4 inhibitors were sufficiently exchangeable for exploratory random-effects synthesis of incident cirrhosis or composite serious liver events. The primary Hartung-Knapp small-sample interval for the pooled estimate was HR 0.85 (95% CI 0.74–0.98); the conventional REML interval was narrower and is reported as a secondary reference estimate (95% CI 0.79–0.93; I²=0%; REML τ²=0.000). Certainty of evidence was rated very low because all included studies were observational, outcome definitions and comparator strategies varied, and residual confounding and healthcare-engagement bias could not be excluded. GLP-1RA use shows a directionally favorable signal for advanced liver outcomes in T2D, but the evidence should be interpreted as hypothesis-supporting rather than definitive causal proof.

**Systematic review registration:**

https://www.crd.york.ac.uk/PROSPERO/view/CRD420261299499, identifier CRD420261299499.

## Introduction

Advanced liver outcomes—including progression to cirrhosis, hepatic decompensation, hepatocellular carcinoma (HCC), liver transplantation, and liver-related mortality—drive excess morbidity and healthcare utilization among patients with type 2 diabetes (T2D) and metabolic dysfunction-associated steatotic liver disease (MASLD).

A rapidly expanding body of real-world evidence (RWE), including studies using target-trial emulations, now addresses GLP-1RA associations with ALOs in diverse populations, including MASLD/T2D, broader T2D cohorts, and ALD. Yet interpretation is challenged by heterogeneity in liver phenotype definitions, comparator selection (active vs non-user), analytic frameworks (new-user design, propensity score, target-trial emulation), and outcome ascertainment (1–7).

Several prior syntheses have examined GLP-1RAs, SGLT2 inhibitors, and liver-related endpoints, including recent network meta-analytic work that ranked drug classes across heterogeneous liver-related outcomes (8–10). However, most syntheses have emphasized biochemical, imaging, histologic, surrogate, or broad composite outcomes rather than advanced clinical liver events, and they have not consistently separated evidence by comparator exchangeability. The unresolved question for endocrinologists is not whether GLP-1RAs improve metabolic or histologic markers, but whether comparative real-world evidence can credibly support a lower risk of cirrhosis, decompensation, HCC, or other advanced liver outcomes.

We therefore synthesized full-text comparative-effectiveness evidence on GLP-1RA-based therapies and advanced liver outcomes in T2D, emphasizing (i) design features that mitigate bias, (ii) comparator choice, (iii) exchangeability of endpoints before pooling, and (iv) conservative interpretation of certainty. The aim was to clarify what current real-world evidence supports, what it cannot support, and which design features future studies must address.

## Methods

### Protocol and reporting

This systematic review was reported using applicable PRISMA 2020 items (11) and uses the MASLD nomenclature as recommended (12). We applied SWiM (Synthesis Without Meta-analysis) and performed exploratory stratified quantitative synthesis only when pooling was clinically and methodologically defensible. Because the eligible evidence consisted exclusively of non-randomized comparative studies, certainty was graded conservatively.

### Protocol deviations from PROSPERO registration

The PROSPERO record listed a broader initial search plan. During finalization of the review question and before quantitative synthesis, the submitted evidence synthesis was narrowed to peer-reviewed comparative real-world cohort studies in adults with T2D reporting advanced clinical liver outcomes. The narrowing was decided prior to screening for inclusion in the pooled stratum, not after viewing effect estimates, and was undertaken to avoid pooling surrogate biochemical markers, imaging outcomes, histologic endpoints, and hard clinical liver events into a single clinically ambiguous summary estimate. We acknowledge that this deviation may have reduced the breadth of evidence captured and may have excluded studies addressing intermediate liver-disease markers. We therefore present this work as a systematic review and exploratory meta-analysis with transparent protocol deviations (**Supplementary Table 1**), while avoiding claims of exhaustive all-database evidence capture.

Heterogeneity was quantified using I² (13) and τ² (14).

Risk of bias was assessed using ROBINS-I (15); synthesis decisions followed SWiM guidance (16); and we recorded the use of multiple imputation for missing covariates where applicable (17).

Planned quantitative synthesis: We predefined stratified random-effects meta-analysis when ≥2 studies addressed the same outcome-comparator question and were sufficiently exchangeable. Where criteria were met, we pooled log-transformed adjusted ratios using a restricted maximum likelihood (REML) random-effects model. Although the primary active-comparator stratum yielded an I² of 0%, this should be interpreted cautiously because heterogeneity statistics have low power when the number of studies is small (k=4). Where criteria were not met, we present study-level adjusted estimates and synthesize qualitatively.

For the primary pooled stratum (k=4), we treated the Hartung-Knapp-Sidik-Jonkman small-sample interval as the primary inferential interval. Because τ² was estimated as 0, the modified Knapp-Hartung/IntHout truncation (HK adjustment with q ≥ 1) was applied to avoid spuriously narrow confidence intervals (18–20). This yielded HR 0.85 with an approximate 95% CI of 0.74–0.98; the conventional REML CI is reported only as a secondary reference estimate (**Supplementary Table 2**). Prediction intervals were not emphasized because τ² was estimated as 0 and the pooled stratum included only four studies, making prediction-interval interpretation unstable.

### Predefined outcome operationalization

To harmonize endpoints across primary studies, we predefined a composite outcome family—”incident cirrhosis or composite serious liver events”-encompassing (i) newly diagnosed cirrhosis and (ii) downstream serious liver events (hepatic decompensation, HCC, and liver-related mortality), following the outcome-coding framework validated in administrative-claims settings (21). Within this operationalized family, studies reporting either incident cirrhosis alone or a composite of these downstream events were considered sufficiently exchangeable for random-effects pooling, provided that other exchangeability criteria (comparator class, new-user design, and follow-up alignment) were met. A predefined endpoint-restricted sensitivity analysis limited to incident-cirrhosis studies only (**Supplementary Table 3**) was used to evaluate the robustness of this operationalization. (Note: this is a subgroup/endpoint-restricted analysis; the conventional leave-one-out omit-one-at-a-time results are reported separately in Supplementary Methods Section 6 and **Supplementary Table 3**).

### Structured exchangeability framework

Before quantitative synthesis, we applied a structured exchangeability framework. Studies were considered eligible for pooling only when they aligned across six prespecified domains: (i) comparator alignment—same clinically relevant comparator class, with preference for active-comparator new-user designs; (ii) outcome-family alignment—incident cirrhosis or composite serious liver events, including hepatic decompensation, HCC, liver transplantation, or liver-related mortality; (iii) design alignment—comparative cohort framework with adjustment for key baseline confounders; (iv) effect-measure compatibility—estimates transformable to the log-relative scale and clinically interpretable as relative event measures; (v) follow-up context—comparable observation windows for the index outcome; and (vi) source-population/estimand independence—no obvious duplicate source-population estimands among studies entering the same pool. Studies with non-exchangeable comparators, materially different estimands, insufficiently aligned endpoints, or likely duplicate source-population estimands were summarized narratively rather than pooled. This framework explains why four GLP-1RA versus DPP-4 inhibitor studies (1, 4, 7, 22) were entered into the primary pool and why studies with non-initiator (5), SGLT2i (6), insulin (2), combination-therapy (23), add-on (24), or within-class (25) comparators were summarized narratively. Study-level application of these criteria is summarized in the SWiM grouping rules in Supplementary Methods Section 3.

### Information sources and study identification

Focused systematic evidence-identification strategy. Candidate studies were identified through PubMed/MEDLINE, Embase, citation chasing, and a PubMed/MEDLINE update through 25 April 2026. This strategy was designed to capture the recent, specialized universe of peer-reviewed comparative-effectiveness studies with advanced liver endpoints in adults with T2D. It was not intended to replace an exhaustive all-database systematic search; this limitation is stated in the protocol-deviation section and in the limitations.

Titles/abstracts were screened for relevance, and full texts were sought for all potentially eligible records using institutional access and publisher sites when available. When full text could not be obtained, the record was retained in the PRISMA accounting and documented as not retrievable with the reason (**Supplementary Table 4**). For transparency, all included studies with DOI-verified citation metadata are cataloged in **Supplementary Table 5**.

### Eligibility criteria

We included full-text, peer-reviewed comparative studies evaluating GLP-1RA initiation (or GLP-1RA-based regimens) in adults with T2D and reporting at least one HLO: cirrhosis incidence/progression, hepatic decompensation events, HCC incidence, liver transplantation, liver-related mortality, or the predefined composite outcome family defined above. We included real-world cohort studies, including studies using target-trial emulations; we excluded small non-comparative series, trials without advanced liver endpoints, and studies limited to intermediate markers.

### Study selection and data extraction

Two reviewers independently assessed eligibility at full text; disagreements were resolved by consensus. Two reviewers independently extracted data using a piloted, standardized form. Discrepancies were resolved by consensus, with arbitration by a third reviewer when needed. We extracted: population (MASLD/CLD/ALD/cirrhosis), design (new-user, active comparator, target-trial), data source, exposure/comparator, follow-up, outcome definitions, effect estimates (HR/RR/IRR as reported), and covariate adjustment strategy. No automation tools were used.

### Risk of bias assessment

Two reviewers assessed ROBINS-I independently; disagreements were resolved by consensus. For non-randomized comparative studies, we assessed risk of bias using ROBINS-I domains (confounding; selection; classification; deviations; missing data; outcome measurement; selective reporting). Study-level judgments are summarized in **Supplementary Table 6** (ROBINS-I) (15) and **Supplementary Figure 1** (ROBINS-I) (15). We recorded whether missing covariates were addressed using multiple imputation or related approaches (17). No automation tools were used.

### Synthesis approach (SWiM)

Given predefined clinical and methodological heterogeneity across (i) liver phenotypes, (ii) comparator strategies, (iii) effect measures, and (iv) outcome definitions, we did not compute pooled estimates across all studies; instead, we performed exploratory random-effects pooling only in predefined strata where ≥2 sufficiently comparable adjusted estimates were available. We report effect directions and magnitudes within comparator strata: (a) GLP-1RA vs DPP-4i; (b) GLP-1RA vs SGLT2i (head-to-head); (c) GLP-1RA-based combination vs monotherapy; (d) within-class: semaglutide vs other GLP-1RAs.

**Figure 1:**
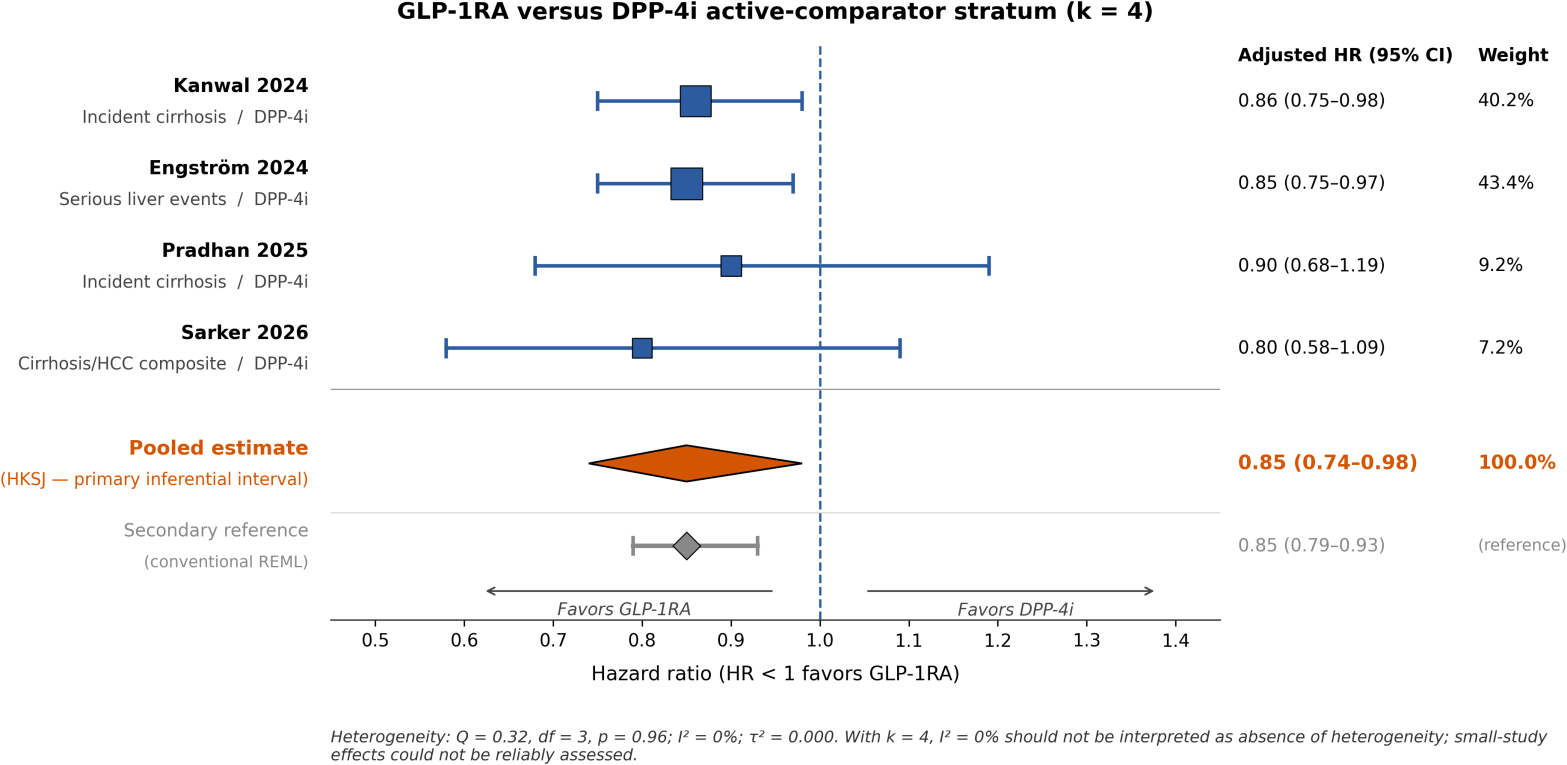
Forest plot of the predefined active-comparator outcome-family stratum (k=4), with Hartung-Knapp inference treated as primary. Four studies (Kanwal 2024, Engström 2024, Pradhan 2025, Sarker 2026) reporting incident cirrhosis or a composite of serious liver events in adults with T2D treated with GLP-1RA versus DPP-4 inhibitors are displayed with study-level endpoint labels to avoid implying complete endpoint identity across studies. The Kanwal 2024 estimate corresponds to the no-baseline-cirrhosis subgroup for incident cirrhosis. The Sarker 2026 estimate shown is the GLP-1RA versus DPP-4i contrast; its GLP-1RA versus SGLT2i contrast was summarized narratively and was not entered into this pool. The primary pooled estimate is HR 0.85 with Hartung-Knapp 95% CI 0.74–0.98; the conventional REML 95% CI 0.79–0.93 is shown as a secondary reference interval. Heterogeneity: Q = 0.32, df=3, p=0.96; I²=0%; τ²=0.000.

**Figure 1** presents the forest plot of the predefined active-comparator stratum and labels the study-level endpoint and comparator to avoid implying full endpoint identity across studies. Pooled estimates, where predefined and appropriate, are reported in the Results.

### Reporting bias assessment

Because quantitative pooling was strictly limited (k=4 for the primary active-comparator stratum) and design-based heterogeneity across the broader evidence base was substantial, we did not perform formal small-study/publication-bias tests [e.g., Egger’s regression (26)]. Furthermore, we refrained from constructing a funnel plot, as such graphical tools lack sufficient statistical power and reliability when k<10. We emphasize risk-of-bias appraisal, comparator alignment, and sensitivity analyses as the primary safeguards against spurious signals.

### Certainty assessment

Certainty ratings were performed independently by two reviewers using GRADE; disagreements were resolved by consensus. We assessed certainty of evidence for each hard-liver outcome using GRADE, starting at low certainty for non-randomized comparative-effectiveness studies and downgrading for risk of bias (ROBINS-I), inconsistency, indirectness, imprecision, and publication bias where applicable. We also computed E-values to assess robustness to unmeasured confounding (27); GRADE summary judgments are provided in **Supplementary Table 7** (28). No automation tools were used.

Design appraisal was informed by RECORD (29) reporting guidance and by causal-inference methods for target-trial emulation, propensity-score analyses, immortal-time bias considerations, and E-values (13, 14, 27, 30, 31).

## Results

### Study selection

**Figure 2:**
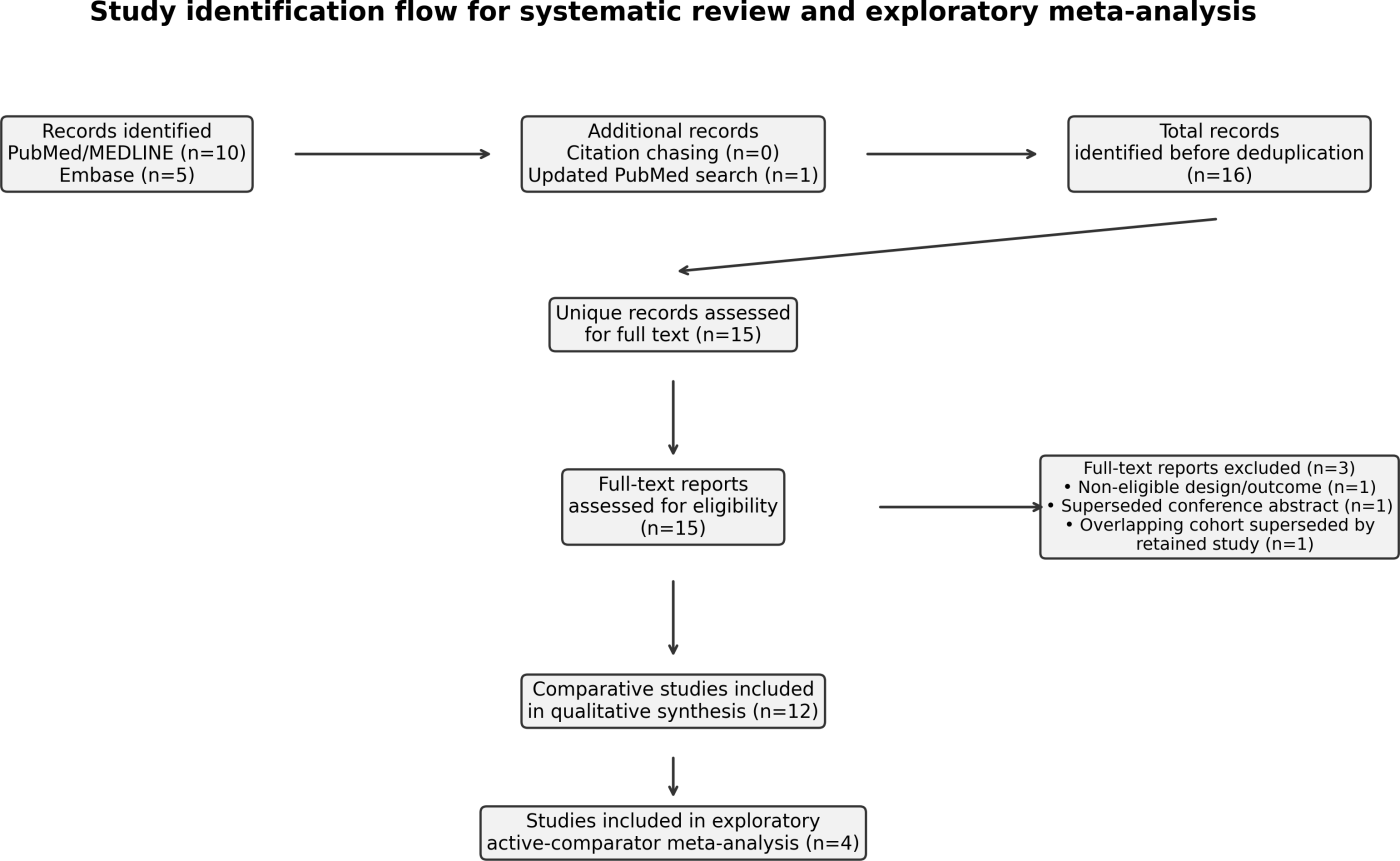
Study identification flow for the submitted systematic review and exploratory meta-analysis. Flow diagram summarizing literature identification, retrieval, eligibility assessment, and inclusion of studies evaluating comparative GLP-1RA-based therapy for advanced liver outcomes in adults with type 2 diabetes. The PROSPERO record specified a broader initial search plan; deviations from the registered protocol are summarized in **Supplementary Table 1**. Exclusion reasons are shown by full-text report, and the retained evidence base comprised 12 comparative real-world cohort studies.

Across all sources, we identified 15 unique records for full-text assessment (**Supplementary Table 8**), including one eligible PubMed/MEDLINE update record published after the structured January 2026 search. Three reports were excluded at full text: one for non-eligible design/outcomes (cross-sectional, no hard liver endpoint); one as a superseded conference abstract without extractable data; and one (32) as an overlapping US administrative cohort whose estimand was superseded by a retained source (Kanwal et al. (1)) with more granular cirrhosis/decompensation adjudication and propensity-score-matched active-comparator design. This left 12 comparative studies for qualitative synthesis (**Figure 2**). Full-text exclusions with reasons are documented in **Supplementary Table 4**, and study-level extracted data are provided in **Supplementary Table 5**.

### Study characteristics

The 12 included studies comprised large database cohorts, including studies using target-trial emulations, spanning MASLD/T2D, broader T2D cohorts, ALD/harmful alcohol use, and cirrhosis populations. Comparator selection varied: active comparators (DPP-4i, SGLT2i, other GLP-1RAs), “other antidiabetic medications,” or non-initiator designs. Key characteristics and primary hard outcomes are summarized in **Table 1**.

**Table 1:** Characteristics of included comparative studies evaluating GLP-1 receptor agonist-based therapies and advanced liver outcomes in adults with type 2 diabetes.

Study	Population/Phenotype	Exposure	Comparator	Primary advanced-liver outcome(s)	Adjusted effect estimate(s)	Absolute risk data
Kanwal 2024	MASLD + T2D	GLP-1RA initiation	DPP-4i (active comparator; propensity-score matched new-user design)	Incident cirrhosis; composite cirrhosis complications; hepatic decompensation; all-cause mortality.	No baseline cirrhosis: incident cirrhosis aHR 0.86 (0.75–0.98). Baseline cirrhosis: composite complications aHR 0.78 (0.59–1.04). Secondary: decompensation aHR 0.75 (0.55–1.01); mortality aHR 0.89 (0.81–0.98).	Not reported
Engström 2024	T2D (Scandinavian)	GLP-1RA use	DPP-4i (active comparator)	Serious liver events (composite); HCC	HR 0.85 (0.75–0.97) for serious liver events; HCC not clearly reduced.	Not reported
Pradhan 2025	T2D (population-based)	GLP-1RA initiation	DPP-4i	Incident cirrhosis	HR 0.90 (0.68–1.19)	Not reported
Sarker 2026	MASLD + T2D (US claims)	Second-line GLP-1RA initiation	DPP-4i and SGLT2i active comparators (pairwise PS-matched cohorts; DPP-4i contrast used in primary pool)	Composite incidence of cirrhosis or HCC over 2 years	GLP-1RA vs DPP-4i: HR 0.80 (0.58–1.09; entered in primary pool); GLP-1RA vs SGLT2i: HR 0.76 (0.55–1.04; narratively summarized only).	Cum. incidence reported; ARR NR.
Wester 2024	CLD + T2D (Swedish registers)	GLP-1RA initiators	Non-initiators (emulated target trial; IPW MSM)	MALO: decompensated cirrhosis, HCC, liver transplant, or MALO-related death	10-year ITT RR 0.91 (0.50–1.32); 10-year PP RR 0.51 (0.14–0.88)	Not reported
Bea 2025	CLD + T2D (East Asian, multi-database)	GLP-1RA use	SGLT2i	Hepatic events	HR 0.93 (0.76–1.14) for SGLT2i vs GLP-1RA (inverted GLP-1RA vs SGLT2i: HR 1.08 [0.88–1.32]).	Not reported
Wu 2025	MASLD/CLD (per study)	GLP-1RA + SGLT2i combination	SGLT2i monotherapy	MALO	HR 0.61 (0.53–0.69)	Not reported
John 2025	Harmful alcohol use (± MASLD)	GLP-1RA initiation	Non-GLP-1RA	Composite liver outcomes; mortality	aHR 0.70 (0.56–0.87) composite; aHR 0.43 (0.37–0.49) mortality	Not reported
Rashid 2025	ALD + T2D	GLP-1RA use	Non-GLP-1RA	Hepatic decompensation	aIRR 0.56 (0.36–0.86)	IR NR; aIRR reported.
Huynh 2023	Cirrhosis + T2D	Metformin + GLP-1RA	Metformin alone	Mortality; hepatic complications incl. HCC	HR 0.44 (0.26–0.74)	Not reported
Wang 2024	HCC-focused T2D cohort	GLP-1RA use	Insulin	HCC incidence; hepatic decompensation	HCC HR 0.20 (0.14–0.31); decompensation HR 0.17 (0.15–0.19)	Not reported
Kuo 2025	Within-class GLP-1RA users	Semaglutide	Other GLP-1RAs	MALO	HR 0.79 (0.66–0.94)	Not reported

ALD, alcohol-associated liver disease; aHR, adjusted hazard ratio; aIRR, adjusted incidence rate ratio; RR, risk ratio; CI, confidence interval; CLD, chronic liver disease; DPP-4i, dipeptidyl peptidase-4 inhibitor; GLP-1RA, glucagon-like peptide-1 receptor agonist; HCC, hepatocellular carcinoma; HR, hazard ratio; ITT, intention-to-treat; PP, per-protocol; MASLD, metabolic dysfunction-associated steatotic liver disease; MALO, major adverse liver outcomes; RWE, real-world evidence; SGLT2i, sodium-glucose cotransporter-2 inhibitor; IPW MSM, inverse probability weighting marginal structural model; T2D, type 2 diabetes. Notes: Study-level definitions of advanced liver outcomes and exposure comparators are reported as described in the original publications. Effect estimates are shown as reported (or converted where applicable) and correspond to the primary adjusted model when available.

### Comparative effectiveness across advanced liver outcomes

#### GLP-1RA vs DPP-4i active-comparator evidence

Across multiple large cohorts, GLP-1RA use was generally associated with lower risk of cirrhosis progression and hepatic events, though estimates varied by phenotype and comparator. In MASLD/T2D, Kanwal et al. (1) reported lower incident cirrhosis (aHR 0.86, 95% CI 0.75–0.98), hepatic decompensation (aHR 0.75, 95% CI 0.55–1.01), and all-cause mortality (aHR 0.89, 95% CI 0.81–0.98) with GLP-1RA initiation versus DPP-4 inhibitor initiation. In a Scandinavian T2D cohort, Engström et al. (4) reported an association with lower serious liver events (HR 0.85, 95% CI 0.75–0.97) using DPP-4i as the active comparator, while HCC risk was not clearly reduced. In a population-based comparison versus DPP-4i, Pradhan et al. (7) found no clear association of GLP-1RA with incident cirrhosis (HR 0.90, 95% CI 0.68–1.19), contrasting with SGLT2i benefit in the same framework. In a target-trial emulation among CLD/T2D, Wester et al. (5) compared GLP-1RA initiators versus non-initiators (inverse probability weighting marginal structural model) and observed consistent but non-definitive favorable effects (10-year intention-to-treat RR 0.91, 95% CI 0.50–1.32), with a stronger per-protocol estimate (RR 0.51, 95% CI 0.14–0.88).

### Stratified meta-analysis (active-comparator stratum)

Within the predefined active-comparator outcome-family stratum (GLP-1RA vs DPP-4 inhibitors for incident cirrhosis or composite serious liver events), four studies (1, 4, 7, 22) were sufficiently exchangeable for exploratory pooling. The primary Hartung-Knapp small-sample interval for the pooled estimate was HR 0.85 (95% CI 0.74–0.98). The conventional REML random-effects model yielded the same point estimate with a narrower secondary interval (95% CI 0.79–0.93; I²=0%; τ²=0.000; Q = 0.32, df=3, p=0.96). This estimate should be read as a directionally consistent comparative signal, not as definitive causal evidence or a single transportable effect across all GLP-1RA populations.

Quantitative extensions that rely on a single global pooled effect (e.g., meta-regression or small-study bias tests) were not performed because most outcomes/comparators remained non-exchangeable and the number of pooled strata with k≥3 was limited.

### analyse Sensitivity s

Two predefined sensitivity analyses were performed for the active-comparator pooled stratum. First, an endpoint-restricted sensitivity analysis limited to studies reporting incident cirrhosis (1, 7) preserved the direction and approximate magnitude of the primary finding (HR 0.87, 95% CI 0.77–0.98). Because this analysis included only two studies, it should be interpreted cautiously as a sensitivity check rather than a confirmatory subgroup analysis. Second, leave-one-out sensitivity analyses showed that omitting any single study did not reverse the direction of association: omitting Kanwal 2024 (1)yielded HR 0.85 (95% CI 0.76–0.95); omitting Engström 2024 (4), HR 0.86 (95% CI 0.77–0.96); omitting Pradhan 2025 (7), HR 0.85 (95% CI 0.78–0.93); omitting Sarker 2026 (22), HR 0.86 (95% CI 0.79–0.94). Given the small number of studies, these diagnostics should be interpreted as checks for gross single-study dominance rather than as definitive evidence of robustness. Detailed sensitivity analyses are provided in **Supplementary Tables 2**, **3**.

### GLP-1RA vs SGLT2i (head-to-head) and GLP-1RA-based combinations

Head-to-head comparisons directly informing therapeutic sequencing were limited and inconclusive. Bea et al. (6) compared SGLT2i versus GLP-1RA and found no clear difference in composite hepatic events (HR 0.93, 95% CI 0.76–1.14 as reported for SGLT2i vs GLP-1RA; inverted GLP-1RA vs SGLT2i direction: HR 1.08, 95% CI 0.88–1.32). Sarker et al. (22) compared second-line GLP-1RA, SGLT2i, and DPP-4i initiators in MASLD/T2D and found no statistically significant 2-year differences across classes, including GLP-1RA versus SGLT2i. These non-significant estimates should not be interpreted as evidence of equivalence or as ruling out clinically relevant differences; rather, the available data indicate that current evidence is insufficient to choose GLP-1RAs over SGLT2 inhibitors solely for liver-event prevention.

### Phenotype-specific evidence (ALD/harmful alcohol use, cirrhosis, HCC-focused cohorts)

In alcohol-related phenotypes, evidence suggested potential benefit for hepatic events and mortality, but confounding risk remained substantial. In harmful alcohol use, John et al. (33) reported an association with lower composite liver-related outcomes (aHR 0.70, 95% CI 0.56–0.87) and lower mortality (aHR 0.43, 95% CI 0.37–0.49) with GLP-1RA. In ALD/T2D, Rashid et al. (3) reported a lower adjusted incidence rate of hepatic decompensation (aIRR 0.56, 95% CI 0.36–0.86).

In cirrhosis/T2D, Huynh et al. (24) reported an association between dual metformin+GLP-1RA therapy versus metformin alone and lower mortality and fewer hepatic complications including HCC (HR 0.44, 95% CI 0.26–0.74); as an add-on therapy contrast, residual confounding (e.g., treatment selection, disease severity) cannot be excluded.

In an HCC-focused cohort, Wang et al. (2) reported an association between GLP-1RA use and lower HCC incidence compared with insulin (HR 0.20, 95% CI 0.14–0.31) and lower hepatic decompensation (HR 0.17, 95% CI 0.15–0.19); because insulin is a less exchangeable comparator in advanced disease, these large effect sizes should be interpreted as associations at high risk of residual confounding rather than as direct pharmacologic effects.

Within class, Kuo et al. (25) reported an association between semaglutide versus other GLP-1RAs and lower major adverse liver outcomes (HR 0.79, 95% CI 0.66–0.94); within-class confounding (dose, adherence, indication) remains possible.

**Supplementary Figure 2** summarizes these phenotype- and comparator-specific estimates.

## Discussion

We undertook this systematic review and exploratory meta-analysis to answer a clinically practical endocrine-hepatic question: whether GLP-1RA-based therapy is associated with lower risk of advanced liver outcomes in comparative real-world cohorts of adults with T2D, and how much confidence clinicians should place in that signal.

### Principal findings and their clinical meaning

The most clinically relevant pattern is not that GLP-1RAs can yet be considered liver-preventive drugs, but that active-comparator real-world evidence shows a consistent, directionally favorable signal for advanced liver outcomes. This matters for endocrinology practice because GLP-1RAs are already prescribed for glycemic control, obesity, and cardiometabolic risk; potential hepatic benefit may inform shared decision-making in patients with T2D, obesity, MASLD, or elevated fibrosis risk, while remaining insufficient as a stand-alone indication.

Absolute effects matter for clinical decisions because baseline liver risk varies widely across populations and designs. Accordingly, we emphasize effect estimates and uncertainty at the study level rather than inferring a single pooled risk reduction.

### Contribution relative to prior syntheses

Prior syntheses are valuable for drug-class ranking across broad liver-related outcomes in MASLD/T2D, but they often combine heterogeneous endpoints, comparators, and evidence types. The contribution of the present review is narrower and deliberately clinical: it prioritizes advanced patient-important liver outcomes, separates evidence by comparator exchangeability, avoids a global pooled estimate across non-exchangeable designs, and treats target-trial emulations and conventional comparative cohorts through a common ROBINS-I, GRADE, E-value, and SWiM framework.

The recent network meta-analysis by Li et al. (10) provides important comparative context by evaluating SGLT2 inhibitors and GLP-1 receptor agonists across liver-related events. Our review should not be read as a competing class-ranking exercise or as proof that GLP-1RAs are liver-preventive drugs. Instead, it asks where the current comparative real-world signal is strongest, where it is weakest, and why head-to-head GLP-1RA versus SGLT2i evidence remains insufficient for therapeutic sequencing.

### Biological plausibility and mechanistic coherence

The observed direction of association is biologically plausible through several converging pathways. First, GLP-1RAs produce sustained weight loss and improvements in insulin resistance, which directly target key drivers of MASLD progression: hepatic *de novo* lipogenesis, adipose inflammation, and lipotoxicity (8, 34). Second, GLP-1RA therapy improves glycemic variability and may reduce oxidative stress and advanced glycation end-product signaling, mechanisms implicated in fibrogenesis and hepatocarcinogenesis (35, 36). Third, GLP-1RAs can reduce systemic and hepatic inflammation via modulation of cytokine profiles, macrophage polarization, and downstream fibrogenic signaling pathways (36, 37). Fourth, GLP-1RAs may influence the gut-liver axis: delayed gastric emptying and appetite modulation alter nutrient flux, and emerging evidence suggests favorable shifts in gut permeability and bile acid signaling may attenuate hepatic inflammation (38). Fifth, improvements in atherometabolic health may indirectly mitigate portal pressure progression and decompensation risk in metabolically driven liver disease contexts (36, 39).

Nevertheless, mechanistic plausibility does not guarantee a hard-outcome effect in heterogeneous real-world populations. Several pathways (e.g., weight loss, insulin sensitivity) are likely necessary but not sufficient, and the “dose” of metabolic improvement required to alter fibrosis trajectory may vary by genotype, baseline fibrosis stage, and co-morbid exposures (alcohol use, viral hepatitis, medications, and socioeconomic factors). This mechanistic nuance aligns with the observed heterogeneity: GLP-1RAs may reduce events more consistently in MASLD-dominant phenotypes than in mixed chronic liver disease populations where etiologic drivers are less responsive to metabolic modulation.

### Reconciling RWE hard outcomes with RCT evidence on liver disease

A recurring challenge in this literature is bridging randomized evidence that emphasizes histologic or imaging endpoints with RWE that focuses on clinical outcomes (decompensation, cirrhosis complications, HCC, liver-related mortality). Randomized trials in MASLD and metabolic dysfunction-associated steatohepatitis (MASH) have shown that GLP-1RAs can improve steatosis and, in some contexts, resolve steatohepatitis; however, effects on fibrosis stage are more variable, and trial durations are often insufficient to capture hard endpoints. RWE, in contrast, observes harder outcomes over longer horizons but is vulnerable to confounding, exposure misclassification, and outcome ascertainment differences. The most coherent synthesis is that GLP-1RAs plausibly shift intermediate biology (weight, insulin resistance, inflammation) and may slow progression sufficiently that hard outcomes decline in some high-risk groups—particularly where baseline risk is high and follow-up is long—yet the observational nature of RWE means that the apparent magnitude is highly sensitive to design quality and comparator choice.

The phase 2 evidence from subcutaneous semaglutide in NASH showed steatohepatitis resolution but an uncertain fibrosis effect (34); this has now been substantively extended by the phase 3 ESSENCE trial (40), which provides direct randomized histologic context. Once-weekly semaglutide 2.4 mg significantly improved steatohepatitis resolution and fibrosis improvement without worsening of MASH at 72 weeks in patients with biopsy-confirmed MASH and F2-F3 fibrosis (40). The subsequent FDA approval of semaglutide for noncirrhotic MASH with moderate-to-advanced fibrosis confirms that GLP-1RA-based hepatic benefit is now supported at the histologic regulatory-endpoint level (41). These findings are biologically upstream of the clinical events examined here—incident cirrhosis, hepatic decompensation, HCC, transplantation, and liver-related mortality—but they do not by themselves prove multi-year hard-outcome prevention. Tirzepatide also improved MASH resolution and fibrosis improvement in the SYNERGY-NASH phase 2 trial, reinforcing a broader incretin-based mechanistic signal while remaining outside the present review’s eligible hard-outcome comparative-effectiveness evidence base (42).

It is also important to consider that RWE “hard outcomes” can reflect composite definitions incorporating diagnostic codes, hospitalization claims, and surrogate proxies for clinical events (21). Such outcomes may be more sensitive than histology to capture clinically relevant progression, but may also be less specific. Misclassification generally biases estimates toward the null when nondifferential, but differential ascertainment (e.g., GLP-1RA users engaging more with healthcare, receiving more imaging, or being monitored more intensively) can bias in either direction. Greater healthcare engagement could increase detection of early cirrhosis or HCC (biasing toward harm) while simultaneously improving management and preventing decompensation (biasing toward benefit). The net direction is therefore context-dependent and reinforces the need for careful outcome definition and sensitivity analyses restricted to validated outcomes.

A related concern is pseudo-replication. Multiple real-world studies may draw from overlapping claims, Veterans Affairs, TriNetX, regional registry, or multi-institutional data environments, even when the final analytic samples and estimands differ. We therefore interpret the consistency of study-level direction as supportive but not as equivalent to replication across fully independent patient populations.

### Heterogeneity and the limits of an “average benefit” estimate

Substantial heterogeneity is a defining feature of this evidence base and should be viewed as clinically informative rather than as a nuisance statistic. Across materially different settings, including baseline liver phenotype (MASLD vs mixed chronic liver disease), comparator choice (DPP-4 inhibitors, SGLT2i, insulin, or broad non-user comparators), healthcare system and coding practices, follow-up duration, and analytic design (target-trial emulation vs conventional adjustment)-study-level estimates are not expected to be constant. For clinicians, this means that “GLP-1RAs are associated with lower liver event rates in several contemporary cohorts” should not be translated into a guaranteed patient-level benefit unless the patient’s context resembles the settings in which benefit was observed.

### Heterogeneity in endpoints and the gap in absolute risk reporting

A critical limitation across the current literature is the widespread underreporting of absolute risk differences or incidence rates. While relative risk reductions (e.g., hazard ratios) were consistently reported, absolute benefit likely depends strongly on baseline fibrosis risk. Without transparent absolute risk metrics, translating these relative benefits into individual clinical decisions remains challenging. For transparency, we provide illustrative absolute risk-reduction scenarios across baseline 5-year event rates assuming the pooled HR 0.85 applied uniformly (**Supplementary Table 9**); these scenarios are explicitly illustrative and should not be interpreted as formal NNT estimates or used for individual clinical decisions without proper risk stratification, a baseline survival function, and competing-risk modeling.

Furthermore, “advanced liver outcomes” encompassed a spectrum of endpoints across studies, ranging from early incident cirrhosis to hepatic decompensation and HCC. The pathophysiological mechanisms by which GLP-1RAs may prevent early fibrotic progression could differ from those mitigating end-stage portal hypertension complications or hepatocarcinogenesis. To address this, we predefined an operationalized composite outcome family (incident cirrhosis or composite serious liver events) with an explicit endpoint-restricted sensitivity analysis; the k=2 incident-cirrhosis-only pooled estimate (HR 0.87, 95% CI 0.77–0.98; **Supplementary Table 3**) preserved the direction and magnitude of the main finding. Nevertheless, pooling across endpoint types should be interpreted with caution, and phenotype-specific effects may remain obscured within composite summaries.

We therefore emphasize three practical strategies to interpret heterogeneity. First, prioritize subgroup-coherent comparisons (active comparator, similar baseline liver risk) over aggregating effects across disparate comparators. Second, focus on transportability: ask whether a given study’s design, comparator, and outcome definition map onto the clinical decision at hand, and treat discordant settings as separate evidence rather than as noise. Third, use influence diagnostics and sensitivity analyses to assess whether the overall pattern is driven by any single high-leverage study. In our synthesis, leave-one-out analyses preserved the directionality of association in the active-comparator stratum, which increases confidence that the signal is not purely an artifact of a single dataset. At the same time, heterogeneity remained substantial at the broader evidence-base level, suggesting that the magnitude is not stable.

### East Asian evidence and transportability

Transportability to East Asian populations deserves explicit attention. Two retained studies are especially relevant: Bea et al. evaluated hepatic events in an East Asian multi-database cohort, and Wu et al. evaluated GLP-1RA plus SGLT2i combination therapy in a Taiwanese setting (6, 23). East Asian MASLD often occurs at lower BMI thresholds and may show different metabolic, genetic, and fibrosis-risk profiles than Western obesity-dominant MASLD. These features make East Asian data a strength of the evidence base, not a peripheral subgroup; however, they also caution against assuming that effect magnitudes from North American or European claims cohorts transport directly to Taiwan or other East Asian health systems. Future studies should report BMI-stratified, fibrosis-stage-stratified, and ethnicity-specific estimates with standardized outcome definitions.

### Causal inference and bias: why the comparator matters

Residual confounding by indication is arguably the dominant threat. GLP-1RAs are preferentially prescribed to patients with obesity, inadequate glycemic control, and higher cardiometabolic risk, but also to patients with better access to specialty care, higher adherence, and more proactive health behaviors. These characteristics correlate with liver outcomes in complex ways. Obesity and metabolic severity generally increase liver risk, which could bias GLP-1RA associations toward harm if inadequately controlled; conversely, healthcare engagement and adherence can bias toward benefit. Frailty, advanced liver disease, or chronic kidney disease may push clinicians away from GLP-1RAs and toward other therapies, which could make GLP-1RA users appear healthier at baseline (healthy-user bias). The net confounding direction cannot be assumed; it must be evaluated through study design features and measured covariate balance.

Active comparator new-user designs are therefore essential. Comparisons against insulin are often non-exchangeable: insulin use frequently marks longer diabetes duration, greater comorbidity burden, and potentially more advanced liver disease, so large “benefits” versus insulin may reflect confounding rather than pharmacology. Comparisons against DPP-4 inhibitors may be more exchangeable in some systems but still vulnerable to channeling by obesity, cardiovascular disease, and formulary restrictions. Comparisons against SGLT2i are particularly informative because GLP-1RA and SGLT2i are both now used earlier in T2D and share indications for cardiovascular and renal protection; however, they may still differ in weight-loss magnitude and in clinical selection based on kidney function, heart failure, and cost. Therefore, comparator-stratified analyses should be considered the primary inferential lens, not optional secondary analyses.

Exposure modeling also matters. GLP-1RA treatment is frequently discontinued, escalated, switched, or combined with SGLT2i or insulin in routine practice. Studies that classify exposure only at baseline may misclassify long-term treatment status, dilute true treatment effects, or introduce immortal-time and adherence-related bias. Future analyses should model GLP-1RA exposure as time-varying and account for persistence, dose escalation, discontinuation, switching, and combination therapy.

Competing risks require equal attention. HCC, hepatic decompensation, liver transplantation, liver-related death, and all-cause mortality are interdependent outcomes. Ordinary Cox models may be less informative when death or transplantation precludes the liver endpoint of interest; competing-risk methods and cause-specific as well as subdistribution estimands should be prespecified when clinically appropriate.

### Quantitative bias analysis and E-values: how robust are the associations?

We assessed the potential impact of unmeasured confounding using the E-value framework (27). As an illustrative benchmark aligned with our primary finding, the pooled HR of 0.85 corresponds to an E-value of 1.63, and the upper Hartung-Knapp confidence limit (HR 0.98) corresponds to an E-value of approximately 1.16. Thus, an unmeasured confounder would need to be associated with both GLP-1RA use and the advanced liver outcome by a risk ratio of at least 1.63 (above and beyond measured covariates) to move the point estimate to the null, whereas the confidence-limit robustness is only modest. The E-value should therefore not be interpreted as evidence of causality; it is a quantitative sensitivity analysis that complements, but does not replace, careful design-based assessment of confounding, comparator exchangeability, healthcare-engagement bias, and differential surveillance. These calculations do not substitute for careful design and confounding control in real-world comparative-effectiveness studies.

### Clinical implications: patient selection, sequencing, and monitoring

If GLP-1RAs do reduce advanced liver outcomes, the most plausible beneficiaries are patients with T2D who have (i) confirmed MASLD/MASH with advanced fibrosis (F2-F3) or compensated cirrhosis, (ii) obesity or substantial insulin resistance, and (iii) an expected treatment horizon of several years. In such patients, GLP-1RAs may simultaneously address weight, glycemic control, cardiovascular risk, and potentially liver progression. Conversely, in patients with low baseline liver risk (early steatosis without fibrosis), the incremental liver benefit is unlikely to justify selection purely on hepatic grounds, though GLP-1RAs may still be chosen for metabolic indications.

Therapeutic sequencing remains a real-world decision problem, but current evidence does not justify choosing a GLP-1RA over an SGLT2 inhibitor solely for liver-event prevention. SGLT2i and GLP-1RAs have overlapping cardiometabolic indications; combination therapy is increasingly used, but comparative liver effectiveness remains uncertain. In practice, treatment choice should remain driven by obesity phenotype, kidney function, heart failure status, cardiovascular risk, tolerability, access, and patient preference. A pragmatic approach is to prioritize GLP-1RA therapy when obesity and weight reduction are central, prioritize SGLT2i when heart failure or chronic kidney disease dominates, consider combination therapy in high-risk cardiometabolic phenotypes if tolerability and access permit, and integrate standard MASLD management in all cases.

Monitoring strategies should reflect the uncertainty and heterogeneity. For patients initiated on GLP-1RAs with advanced fibrosis, monitoring for weight trajectory, glycemic control, and liver biochemistry is routine, but hard-outcome prevention requires attention to cirrhosis surveillance: HCC surveillance adherence, variceal screening in cirrhosis, and management of portal hypertension. Importantly, clinicians should avoid over-attributing improvements (or lack thereof) to the GLP-1RA, given the multifactorial determinants of liver outcomes.

### Costs, adherence, and real-world feasibility

GLP-1RAs are expensive and often limited by access barriers, supply constraints, and gastrointestinal adverse effects. Long-term adherence is critical; discontinuation can attenuate weight loss and potentially erode any liver benefit. Therefore, even if a relative risk reduction is present, real-world effectiveness will be strongly shaped by persistence and adherence. Economic considerations are particularly salient when the expected absolute liver event reduction is small. In many healthcare systems, the strongest rationale for GLP-1RA use remains cardiometabolic risk reduction and weight management; any liver benefit may be best conceptualized as an added value that could shift the decision in borderline cases, rather than as a primary indication by itself (until hard-outcome RCT evidence emerges).

### Strengths of this review

Several strengths merit emphasis. First, we focused on advanced liver outcomes rather than surrogate endpoints, which aligns with patient-important outcomes and policy decisions. Second, we explicitly highlighted comparative-effectiveness designs and target-trial emulation design principles, which are increasingly central to credible RWE inference. Third, we applied structured risk-of-bias assessment for non-randomized studies and integrated certainty considerations via GRADE and E-value analyses. Fourth, our synthesis prioritizes interpretability: rather than presenting a single summary estimate as definitive, we emphasize heterogeneity, design-quality gradients, and setting-specific transportability. Finally, we provide a clinically oriented framing for how these data might inform patient selection and sequencing decisions, while transparently acknowledging uncertainty.

### Limitations

This review has several important limitations that directly affect interpretation. First, the review scope was focused and did not complete every database listed in the broader PROSPERO record, and the April 2026 update was a targeted PubMed/MEDLINE update rather than a complete repeat search of all databases. We therefore avoid claiming exhaustive evidence capture, and this work should be interpreted as a focused systematic review and exploratory meta-analysis rather than an exhaustive mapping of all GLP-1RA liver-related evidence.

Second, all included studies were observational and remain vulnerable to residual confounding, confounding by indication, healthy-user bias, healthcare-engagement bias, and differential surveillance. GLP-1RA users may differ from comparator groups in obesity severity, access to specialist care, adherence, imaging frequency, liver surveillance, and preventive-care behaviors, and these factors may bias estimates in either direction. The E-value analysis indicates only modest robustness of the upper confidence limit to unmeasured confounding and should not be interpreted as evidence of causality.

Third, exposure heterogeneity was substantial. Included studies may have captured different GLP-1RA agents (e.g., semaglutide, liraglutide, dulaglutide), doses, titration patterns, persistence, discontinuation, switching, adherence, and combination therapy. Most studies did not consistently report agent-specific effects, cumulative exposure, or time-varying treatment status. The pooled estimate should therefore not be interpreted as applying equally to all GLP-1RA agents, doses, or treatment patterns.

Fourth, outcome definitions were not identical across studies. Endpoints labeled as advanced liver outcomes ranged from incident cirrhosis to hepatic decompensation, HCC, and composite serious liver events. Although we used a predefined outcome-family framework and an endpoint-restricted sensitivity analysis (HR 0.87, 95% CI 0.77–0.98), pooling across related but non-identical outcomes remains an important interpretive limitation.

Fifth, the exploratory meta-analysis included only four studies. With k=4, the observed I²=0% should not be interpreted as absence of heterogeneity; rather, statistical heterogeneity was not detected within a small and narrowly defined stratum, and small-study effects could not be reliably assessed. Clinical heterogeneity in population phenotype, outcome definitions, follow-up duration, and data source remained relevant.

Sixth, potential overlap among real-world data sources cannot be excluded. Some included studies used large claims, registry, Veterans Affairs, TriNetX, or multi-institutional datasets, and overlapping underlying patient populations are possible even when the final analytic samples and estimands differ. Consistency in the direction of estimates should therefore be interpreted as supportive directional agreement rather than as fully independent replication.

These limitations justify the very-low GRADE certainty rating despite the consistency of the direction of effect, and they support interpreting the findings as hypothesis-supporting rather than practice-changing.

### Future research directions

Future work should prioritize:

Harmonized target-trial emulations using common eligibility criteria, comparator definitions, outcome ascertainment, time-varying exposure modeling, and prespecified competing-risk estimands to improve cross-study comparability.

Head-to-head comparative studies against other liver-protective agents (particularly SGLT2i) with sufficient follow-up and standardized hard endpoints.

Phenotype-stratified analyses (e.g., MASLD, ALD/harmful alcohol use, cirrhosis stage) to identify subgroups most likely to benefit and to reduce heterogeneity driven by case-mix.

Mechanistic and biomarker-anchored studies linking metabolic changes (weight loss, glycemic improvement, inflammation) to longitudinal fibrosis progression and clinical events.

Comparative-effectiveness evaluation of tirzepatide (a dual GIP/GLP-1 receptor agonist) for advanced liver outcomes in adults with T2D, particularly head-to-head against established GLP-1RAs such as semaglutide, given its rapidly expanding clinical use.

Dedicated real-world studies in Latin American, African, and South Asian populations to address generalizability gaps in the existing evidence base.

Routine reporting of absolute risks, incidence rates, risk differences, discontinuation rates, dose escalation, and add-on therapy patterns so that relative associations can be translated into clinically meaningful absolute-benefit scenarios without overstating treatment effects.

## Conclusions

In conclusion, comparative real-world evidence suggests that GLP-1RA use is associated with a directionally lower risk of advanced liver outcomes in adults with T2D, particularly in DPP-4 inhibitor active-comparator settings. However, the evidence remains observational, heterogeneous, and of very low certainty. GLP-1RAs should not be presented as proven liver-preventive therapy on the basis of current evidence; rather, possible hepatic benefit may be considered as a secondary, hypothesis-supporting factor when GLP-1RAs are otherwise clinically indicated for glycemic control, obesity, or cardiometabolic risk.

What this review cannot conclude. The present synthesis cannot determine whether GLP-1RAs causally prevent cirrhosis, hepatic decompensation, HCC, liver transplantation, or liver-related mortality. It cannot determine whether one GLP-1RA agent, dose, or treatment duration is superior to another for liver outcomes, nor whether GLP-1RAs should be prioritised over SGLT2 inhibitors solely for liver-event prevention. The pooled estimate does not establish transportability to populations not represented in the included cohorts, including individuals with low baseline fibrosis risk, individuals with active alcohol use, and East Asian lean-MASLD phenotypes that are under-represented in current real-world evidence. These questions require adequately powered, long-term, head-to-head comparative studies with standardised outcome definitions, time-varying exposure modeling, competing-risk methods, and absolute-risk reporting.

These findings should guide cautious treatment discussions and trial prioritization, not immediate practice-changing claims.

## Data Availability

The original contributions presented in the study are included in the article/supplementary material. Further inquiries can be directed to the corresponding author.
