# Supplementary material for: Glucagon-like peptide-1 receptor agonists and advanced liver outcomes in type 2 diabetes: a systematic review and exploratory meta-analysis
Source: Front Endocrinol (Lausanne). 2026 Jun 2;17:1874720. doi: 10.3389/fendo.2026.1874720 (PMC13268877; doi:10.3389/fendo.2026.1874720)
Supplement: Supplementary file 1 [file DataSheet1.docx]

Supplementary Methods

GLP-1 receptor agonists and advanced liver outcomes in type 2 diabetes: supplementary methods for a systematic review and exploratory meta-analysis

1. Search Strategy and Information Sources

Systematic review scope and focused evidence-identification strategy. Candidate studies were identified through a focused literature-identification strategy appropriate for a recent, specialized universe of comparative-effectiveness studies with advanced liver endpoints in adults with T2D. The submitted review used PubMed/MEDLINE and Embase, citation chasing, and a PubMed/MEDLINE update through 25 April 2026. The scope is intentionally described as focused and should not be interpreted as an exhaustive all-database systematic review.

PubMed/MEDLINE search string

Protocol clarification: The PROSPERO record listed a broader initial search plan. For the submitted manuscript, the review was finalized as a systematic review and exploratory meta-analysis of peer-reviewed comparative real-world cohort studies with advanced liver outcomes in adults with T2D. This narrowing was adopted to avoid mixing surrogate liver outcomes with advanced clinical outcomes and is reported as a protocol deviation.

("glucagon-like peptide-1 receptor agonist*" OR "GLP-1 receptor agonist*" OR "GLP-1RA"

OR semaglutide OR liraglutide OR dulaglutide OR exenatide OR tirzepatide)

AND ("liver" OR "hepatic" OR "cirrhosis" OR "hepatocellular carcinoma"

OR "decompensation" OR "steatotic liver disease" OR "MASLD" OR "NAFLD"

OR "NASH" OR "MASH" OR "alcohol-associated liver")

AND ("type 2 diabetes" OR "T2D" OR "diabetes mellitus")

AND ("cohort" OR "real-world" OR "target-trial" OR "emulation"

OR "comparative" OR "propensity")

Filters: English-language human studies; publication date: 2020/01/01 to 2026/04/25. No full-text availability filter was applied in the final PubMed/MEDLINE update; all full-text exclusions are documented in Supplementary Table S4.

Embase search string

('glucagon like peptide 1 receptor agonist'/exp OR 'semaglutide'/exp

OR 'liraglutide'/exp OR 'dulaglutide'/exp OR 'exenatide'/exp OR 'tirzepatide'/exp)

AND ('liver disease'/exp OR 'liver cirrhosis'/exp OR 'hepatocellular carcinoma'/exp

OR 'hepatic decompensation' OR 'steatotic liver disease' OR 'fatty liver'/exp)

AND ('non insulin dependent diabetes mellitus'/exp OR 'type 2 diabetes')

AND ('cohort study'/exp OR 'real world evidence' OR 'target-trial' OR 'comparative effectiveness')

AND [english]/lim AND [2020-2026]/py (structured Embase search conducted 20 Jan 2026)

Note: Tirzepatide (a dual GIP/GLP-1 receptor agonist) was included as a sensitivity term. No eligible comparative-effectiveness studies of tirzepatide reporting advanced liver outcomes in adults with T2D met inclusion criteria.

2. Predefined Outcome Operationalization

To harmonize endpoints across primary studies, we predefined a composite outcome family of "incident cirrhosis or composite serious liver events" prior to data extraction. This outcome family was operationalized to encompass: (a) newly diagnosed cirrhosis based on clinical coding, fibrosis staging, or hepatologist diagnosis; and (b) downstream serious liver events, including hepatic decompensation (ascites, hepatic encephalopathy, variceal bleeding, spontaneous bacterial peritonitis), HCC incidence, liver transplantation, and liver-related mortality. This operationalization follows the outcome-coding framework validated in administrative-claims settings by Goldberg et al. (Pharmacoepidemiol Drug Saf. 2012;21:765-769; main text reference 21).

Studies reporting either incident cirrhosis alone or a composite of downstream serious liver events were considered sufficiently exchangeable for random-effects pooling within this outcome family, provided other exchangeability criteria (comparator class, new-user design, and follow-up alignment) were concurrently met. A predefined sensitivity analysis restricted to incident-cirrhosis-only studies (i.e., excluding composite-outcome studies) was conducted to evaluate directional consistency, not to validate the composite endpoint.

3. SWiM Grouping Rules (Comparator Strata)

Studies were grouped for narrative synthesis and exploratory quantitative pooling according to the following predefined comparator strata:

- (a) GLP-1RA vs DPP-4 inhibitors: Kanwal 2024, Engström 2024, Pradhan 2025, and Sarker 2026 contributed active-comparator estimates using DPP-4 inhibitor comparators. Sarker's GLP-1RA versus SGLT2i contrast was summarized narratively in the head-to-head stratum and was not entered into the primary pool.
- (b) GLP-1RA vs SGLT2 inhibitors (head-to-head): Direct comparisons between GLP-1RA and sodium-glucose cotransporter 2 inhibitors. Bea 2025 was classified in this stratum (reported as SGLT2i vs GLP-1RA for composite hepatic events: HR 0.93, 95% CI 0.76–1.14; inverted GLP-1RA vs SGLT2i: HR 1.08, 95% CI 0.88–1.32; not pooled).
- (c) GLP-1RA-based combination vs monotherapy: Studies evaluating GLP-1RA in combination with other agents (e.g., SGLT2i) versus monotherapy. Wu 2025 was classified here (GLP-1RA + SGLT2i vs SGLT2i monotherapy).
- (d) Within-class comparisons: Semaglutide vs other GLP-1RAs. Kuo 2025 was classified here.

Exploratory random-effects meta-analysis was performed only in stratum (a), where k=4 studies reported adjusted time-to-event estimates under the predefined composite outcome family. Effect directions and magnitudes were reported within each stratum using direction-of-effect synthesis and study-level adjusted estimates.

4. ROBINS-I Risk-of-Bias Appraisal

Risk of bias for each included non-randomized comparative study was assessed using ROBINS-I across seven domains: (1) bias due to confounding; (2) bias in selection of participants; (3) bias in classification of interventions; (4) bias due to deviations from intended interventions; (5) bias due to missing data; (6) bias in measurement of outcomes; (7) bias in selection of the reported result. Domain-level and overall judgments (low / moderate / serious / critical) are summarized in Supplementary Table S6.

For studies employing multiple imputation for missing covariates, domain 5 (missing data) was adjusted accordingly. Two reviewers performed ROBINS-I assessments independently; disagreements were resolved by consensus.

5. Reference Integrity and Exclusion Documentation

For each included study, DOI and PubMed indexing were re-checked against PubMed and publisher records. PMID values are provided only when PubMed indexing was confirmed; records without confirmed PubMed indexing at the audit date are marked as not available rather than assigned a forced identifier. The corrected citation audit is provided in Supplementary Table S5.

Elsaid MI, et al. (Aliment Pharmacol Ther. 2024) was excluded at full-text review as an overlapping US administrative cohort whose estimand was superseded by the retained source (Kanwal 2024, JAMA Intern Med), which uses more granular cirrhosis/decompensation endpoint adjudication and a propensity-score-matched active-comparator design. Elsaid 2024 is listed in Supplementary Table S4 with this expanded exclusion reason.

6. Meta-analysis Technical Details

Exploratory random-effects meta-analysis was conducted using restricted maximum likelihood (REML) to estimate the between-study variance (τ²). Log-transformed hazard ratios (ln[HR]) and their standard errors (SE = [ln(UCL) - ln(LCL)] / [2 x 1.96]) were used as input for the four studies in the active-comparator stratum (Kanwal 2024, Engström 2024, Pradhan 2025, Sarker 2026).

Although the observed I² was 0% (Q=0.32, df=3, p=0.96) and τ² was estimated as 0.000, a random-effects model was predefined to provide a conservative analytic framework that acknowledges potential between-study variation. With k=4, I² has low power to detect true heterogeneity; therefore, the pooled estimate is interpreted as exploratory and is considered alongside leave-one-out sensitivity analysis and comparator-specific qualitative synthesis.

Prediction intervals were not reported because τ² was estimated as 0 with k=4 (insufficient degrees of freedom for a reliable prediction interval). For studies reporting the comparator in the opposite direction (e.g., SGLT2i vs GLP-1RA in Bea 2025), the estimate was not inverted for pooling; Bea 2025 was classified in the head-to-head stratum, presented in both the original and inverted directions for interpretability, and not included in the primary active-comparator pooled estimate.

Sensitivity analyses: (a) Leave-one-out analyses (Supplementary Table S3): omitting Kanwal: pooled HR 0.85 (95% CI 0.76–0.95); omitting Engström: pooled HR 0.86 (95% CI 0.77–0.96); omitting Pradhan: pooled HR 0.85 (95% CI 0.78–0.93); omitting Sarker: pooled HR 0.86 (95% CI 0.79–0.94). Predefined incident-cirrhosis-only analysis (Kanwal + Pradhan, excluding composite endpoints): pooled HR 0.87 (95% CI 0.77–0.98), demonstrating directional consistency but not validating the composite endpoint. (b) Fixed-effect (inverse-variance) model (Supplementary Table S2): pooled HR 0.85 (95% CI 0.79–0.93). (c) Hartung-Knapp-Sidik-Jonkman primary small-sample interval with modified Knapp-Hartung/IntHout truncation (HK adjustment with q ≥ 1; main text references 15-17): pooled HR 0.85 (approximate 95% CI 0.74–0.98). The conventional REML interval (95% CI 0.79–0.93) is retained only as a secondary reference estimate.

Absolute risk translation (Supplementary Table S9): The pooled relative HR was applied to illustrative baseline 5-year event-rate scenarios (2%, 5%, 10%, 15%, 20%) to derive approximate absolute risk reductions under simplifying assumptions. These scenarios should not be interpreted as formal treatment-number estimates because HRs from observational time-to-event analyses do not directly translate into patient-level absolute benefit without a baseline survival function, competing-risk structure, and treatment-persistence assumptions.

All analyses were conducted in R (version 4.3.x; R Foundation for Statistical Computing, Vienna, Austria) using the metafor package (version 4.x).
