# Supplementary material for: Glucagon-like peptide-1 receptor agonists and advanced liver outcomes in type 2 diabetes: a systematic review and exploratory meta-analysis
Source: Front Endocrinol (Lausanne). 2026 Jun 2;17:1874720. doi: 10.3389/fendo.2026.1874720 (PMC13268877; doi:10.3389/fendo.2026.1874720)

# Supplementary Figures

Supplementary Figure S1. ROBINS-I risk-of-bias heatmap for included non-randomized comparative studies. L, low risk; M, moderate risk; S, serious risk; C, critical risk; NI, no information.


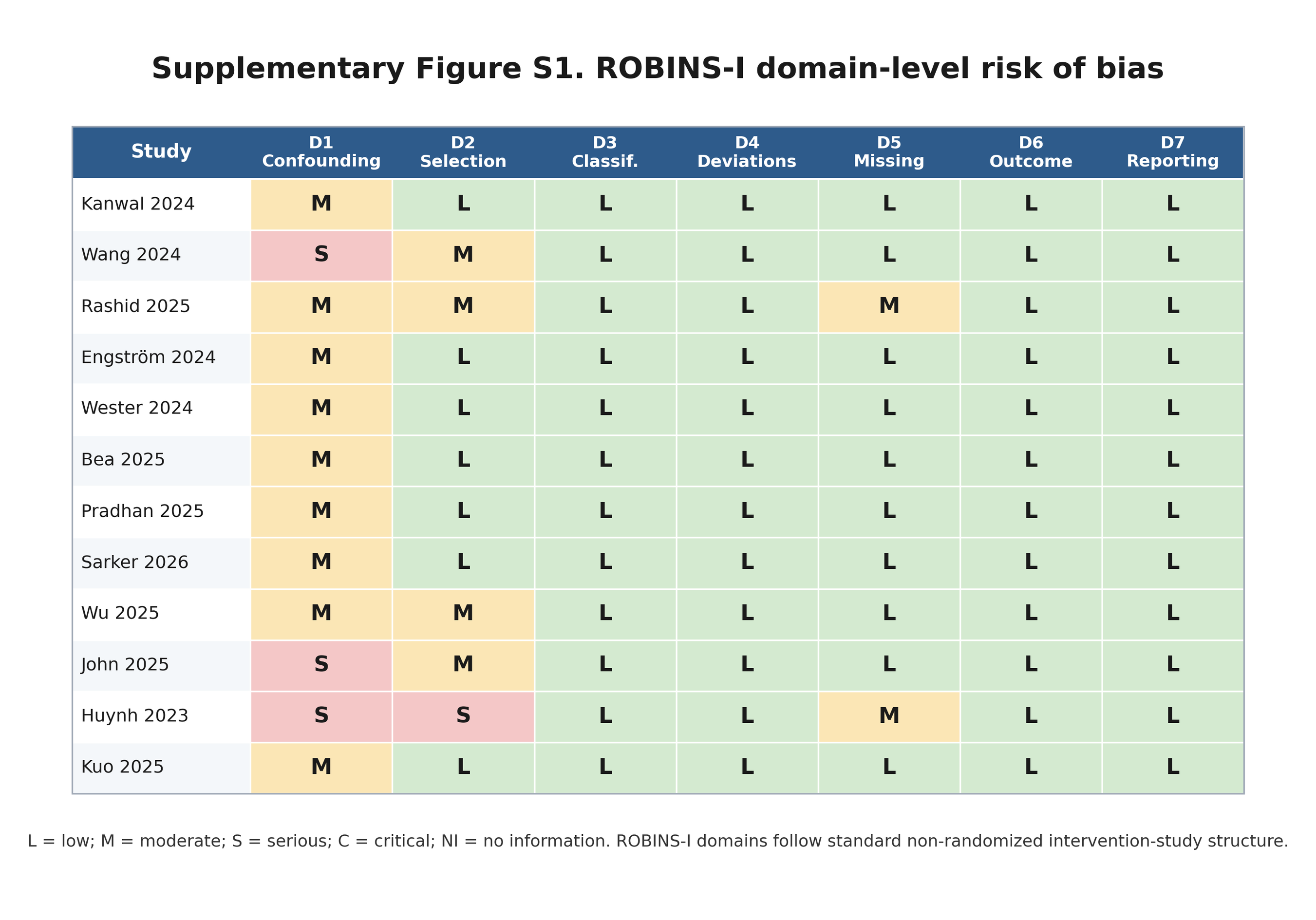


Supplementary Figure S2. Evidence map by phenotype and comparator stratum.


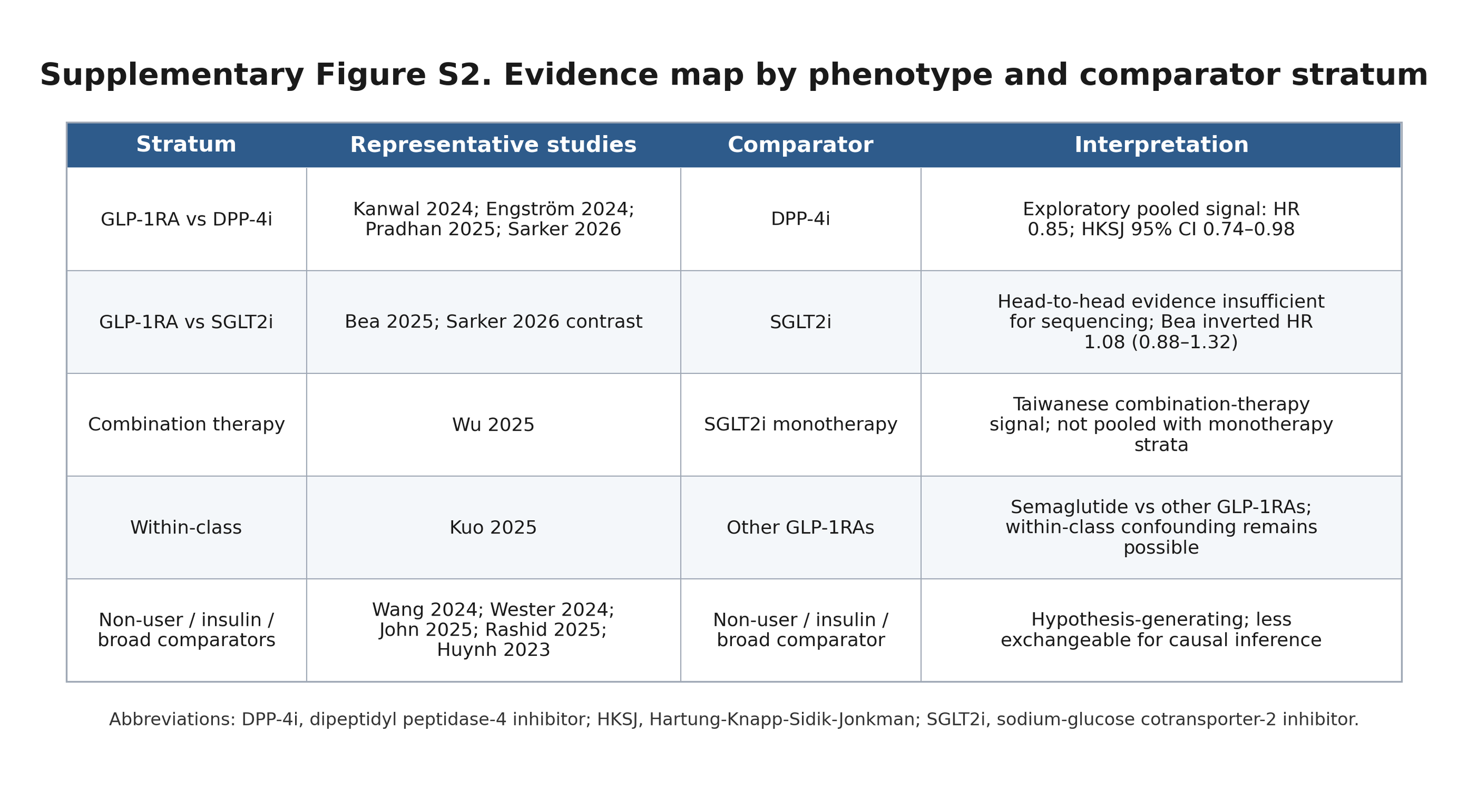

Supplement: Supplementary file 2 [file DataSheet2.docx]
