# Supplementary material for: Glucagon-like peptide-1 receptor agonists and advanced liver outcomes in type 2 diabetes: a systematic review and exploratory meta-analysis
Source: Front Endocrinol (Lausanne). 2026 Jun 2;17:1874720. doi: 10.3389/fendo.2026.1874720 (PMC13268877; doi:10.3389/fendo.2026.1874720)
Supplement: Supplementary file 4 [file DataSheet4.docx]

**PRISMA 2020 Checklist**

GLP-1 receptor agonists and advanced liver outcomes in type 2 diabetes: systematic review of comparative real-world cohort studies

Revised version for Frontiers in Endocrinology, May 2026; v77 revision after peer review

v77 revision after peer review. Key changes: focused evidence-synthesis scope is stated consistently; protocol deviations from the broader registered search plan are disclosed in Supplementary Table S1; study-identification flow is framed without overclaiming exhaustive PRISMA completeness; quantitative synthesis is limited to a predefined exploratory active-comparator outcome-family stratum; HKSJ small-sample inference is treated as primary; and final citation/cover-letter consistency checks were completed.

| **Section** | **Item** | **Checklist item (PRISMA 2020) and final compliance note** | **Location in manuscript** |
| --- | --- | --- | --- |
| TITLE | 1 | Identify the report as a systematic review. [The report is identified as a systematic review with exploratory meta-analysis.] | Title page; Abstract |
| ABSTRACT | 2 | See the PRISMA 2020 for Abstracts checklist. [Abstract describes the focused evidence-identification strategy and prespecified outcome-family operationalization.] | Abstract |
| INTRO | 3 | Rationale: Describe the rationale for the review in the context of existing knowledge. | Introduction |
| INTRO | 4 | Objectives: Provide an explicit statement of the objective(s) or question(s) the review addresses. | Introduction |
| METHODS | 5 | Eligibility criteria: Specify the inclusion and exclusion criteria for the review. | Methods - Eligibility criteria |
| METHODS | 6 | Information sources: Specify all databases, registers, websites, organisations, reference lists and other sources searched or consulted to identify studies. [Information sources and update search are explicitly reported.] | Methods - Information sources; Supp Methods S1 |
| METHODS | 7 | Search strategy: Present the full search strategies for all databases, registers and websites, including any filters and limits used. [Full PubMed/MEDLINE and Embase strings are reported in Supplementary Methods S1; focused scope is transparently disclosed.] | Supp Methods S1 |
| METHODS | 8 | Selection process: Specify the methods used to decide whether a study met the inclusion criteria of the review, including how many reviewers screened each record and full-text, and whether they worked independently. | Methods - Study selection |
| METHODS | 9 | Data collection process: Specify the methods used to collect data from reports, including how many reviewers collected data from each report, whether they worked independently, and any processes for obtaining or confirming data from study investigators. | Methods - Data extraction |
| METHODS | 10a | Data items: List and define all outcomes for which data were sought. [Prespecified outcome family is defined in Methods.] | Methods - Outcome operationalization |
| METHODS | 10b | Data items: List and define all other variables for which data were sought (e.g., participant and intervention characteristics, funding sources). | Methods - Data extraction; Table 1 |
| METHODS | 11a | Study risk of bias assessment: Specify the methods used to assess risk of bias in the included studies, including details of the tool(s) used. | Methods - Risk of bias; Table S6 |
| METHODS | 11b | Study risk of bias assessment: Specify the methods used to assess risk of bias in any reports that investigated the effects of multiple interventions. | Methods - Risk of bias |
| METHODS | 12 | Effect measures: Specify for each outcome the effect measure(s) (e.g. risk ratio, mean difference) used in the synthesis or presentation of results. | Methods - Synthesis; Table 1 |
| METHODS | 13a | Synthesis methods: Describe the processes used to decide which studies were eligible for each synthesis (e.g. tabulating the study intervention characteristics and comparing against the planned groups for each synthesis). | Methods - SWiM; Supp Methods S2-S3 |
| METHODS | 13b | Synthesis methods: Describe any methods required to prepare the data for presentation or synthesis, such as handling of missing summary statistics, or data conversions. | Methods - Meta-analysis; Supp Methods S6 |
| METHODS | 13c | Synthesis methods: Describe any methods used to tabulate or visually display results of individual studies and syntheses. | Figure 2; Supp Figure S2 |
| METHODS | 13d | Synthesis methods: Describe any methods used to synthesise results and provide a rationale for the choice(s). [REML random-effects synthesis is limited to the prespecified active-comparator stratum; SWiM is used for remaining strata.] | Methods - Synthesis approach; Supp Methods S6 |
| METHODS | 13e | Synthesis methods: Describe any methods used to explore possible causes of heterogeneity among study results (e.g. subgroup analysis, meta-regression). | Methods - SWiM; Supp Tables S2-S3 |
| METHODS | 13f | Synthesis methods: Describe any sensitivity analyses conducted to assess robustness of the synthesised results. | Methods - Supp Methods S6; Table S2-S3 |
| METHODS | 14 | Reporting bias assessment: Describe any methods used to assess risk of bias due to missing results in a synthesis (arising from reporting biases). [final: Rationale for not performing Egger test or funnel plot stated explicitly in Methods] | Methods - Reporting bias |
| METHODS | 15 | Certainty assessment: Describe any methods used to assess certainty (or confidence) in the body of evidence for an outcome. | Methods - Certainty; Supp Table S7 |
| RESULTS | 16a | Study selection: Describe the results of the search and selection process, including the number of records identified in each database and those selected for inclusion, ideally using a flow diagram. [final: Figure 1 PRISMA regenerated with targeted identification disclosure and Elsaid exclusion expanded] | Results - Study selection; Figure 1 |
| RESULTS | 16b | Study selection: Cite studies that might appear to meet the inclusion criteria but which were excluded, and explain why they were excluded. | Table S4 (expanded Elsaid exclusion reason) |
| RESULTS | 17 | Study characteristics: Cite each included study and present its characteristics. | Table 1; Results - Study characteristics |
| RESULTS | 18 | Risk of bias in studies: Present assessments of risk of bias for each included study. | Table S6; Supp Figure S1 |
| RESULTS | 19 | Results of individual studies: For all outcomes, present, for each study: (a) summary statistics for each group (if appropriate) and (b) an effect estimate and its precision. | Table 1; Results section |
| RESULTS | 20a | Results of syntheses: For each synthesis, briefly summarise the characteristics and risk of bias among contributing studies. | Results - Stratified meta-analysis |
| RESULTS | 20b | Results of syntheses: Present results of all statistical syntheses conducted. [final: Primary HKSJ pooled HR 0.85 (95% CI 0.74-0.98); secondary REML 0.79-0.93; endpoint-restricted and leave-one-out in main-text Results and Table S3] | Results; Figure 2; Table S2-S3 |
| RESULTS | 20c | Results of syntheses: Present results of all investigations of possible causes of heterogeneity among study results. | Results; Table S2 |
| RESULTS | 20d | Results of syntheses: Present results of all sensitivity analyses conducted to assess the robustness of the synthesised results. | Table S3; Supp Methods S6 |
| RESULTS | 21 | Reporting biases: Present assessments of risk of bias due to missing results (arising from reporting biases) for each synthesis assessed. | Methods - Reporting bias (rationale stated) |
| RESULTS | 22 | Certainty of evidence: Present assessments of certainty (or confidence) in the body of evidence for each outcome assessed. | Table S7 (GRADE - Very Low for all outcomes) |
| DISCUSSION | 23a | Discussion: Provide a general interpretation of the results in the context of other evidence. | Discussion |
| DISCUSSION | 23b | Discussion: Discuss any limitations of the evidence included in the review. | Discussion - Limitations |
| DISCUSSION | 23c | Discussion: Discuss any limitations of the review processes used. [final: Targeted identification strategy limitation explicitly acknowledged] | Discussion - Limitations |
| DISCUSSION | 23d | Discussion: Discuss implications of the results for practice, policy, and future research. | Discussion - Clinical implications; Future research |
| OTHER | 24 | Registration and protocol: Provide registration information for the review, including register name and registration number, or state that the review was not registered. [PROSPERO CRD420261299499] | Title page; Methods - Protocol |
| OTHER | 25 | Support: Describe sources of financial or other support for the review, and the role of the funders or sponsors in the review. [No external funding received] | Declarations - Funding |
| OTHER | 26 | Competing interests: Declare any competing interests of review authors. | Declarations - COI |
| OTHER | 27 | Availability of data, code and other materials: Report which of the following are publicly available and where they can be found: template data collection forms; data extracted from included studies; data used for all analyses; analytic code; any other materials used in the review. | Declarations - Data availability; Supp Materials |

*Adapted from: Page MJ, et al. The PRISMA 2020 statement: an updated guideline for reporting systematic reviews. BMJ. 2021;372:n71. doi:10.1136/bmj.n71. For more information, visit http://www.prisma-statement.org/*
